# Burn Defect and Phenol Prediction for Flavoured Californian-Style Black Olives Using Digital Sensors

**DOI:** 10.3390/foods12071377

**Published:** 2023-03-24

**Authors:** Gema Cascos, Juan Diego Barea-Ramos, Ismael Montero-Fernández, Antonio Ruiz-Canales, Jesús Lozano, Daniel Martín-Vertedor

**Affiliations:** 1 Technological Institute of Food and Agriculture (CICYTEX-INTAEX), Junta of Extremadura, Avda. Adolfo Suárez s/n, 06007 Badajoz, Spain; 2 Department of Chemical Engineering and Physical Chemistry, Area of Chemical Engineering, Faculty of Sciences, University of Extremadura, Avda. de Elvas, s/n, 06006 Badajoz, Spain; 3 Engineering Department, Miguel Hernández University of Elche, Politechnic High School of Orihuela, 03312 Elche, Spain; 4 Industrial Engineering School, University of Extremadura, 06006 Badajoz, Spain

**Keywords:** sterilization treatment, stuffed olives, sensory analysis, burn defect, E-nose, phenols

## Abstract

Californian-style black olives can undergo different chemical changes during the sterilization process that can affect their sensory and phenol characteristics. Thus, these olives were stuffed with flavoured hydrocolloids and submitted to different thermal sterilization treatments to assess sensory categories. The triangular test indicated that the panellists were able to discriminate between samples from different categories according to their aromas with more than 85% success. The results indicated that the negative aroma detected by tasters was related to burn defects. The highest level of defects was found in standard olives, while the lowest was identified in the extra category. Furthermore, olives submitted to the lowest thermal sterilization treatment (extra) presented significantly higher phenol profile content, such as for hydroxytyrosol, tyrosol, oleuropein and procyanidin B1. The electronic nose (E-nose) discriminated between samples from different categories according to the specific aroma (PC1 = 82.1% and PC2 = 15.1%). The PLS-DA classified the samples with 90.9% accuracy. Furthermore, the volatile organic compounds responsible for this discrimination were creosol, copaene, benzaldehyde and diallyl disulphide. Finally, the models established by the PLS analysis indicated that the E-nose could predict olives according to their aroma and total phenol profile $(R_{CV}^{2}$ values were 0.89 and 0.92, respectively). Thus, this device could be used at the industrial level to discriminate between olives with different sensory aromas to determine those with the highest quality.

## 1. Introduction

Table olives (*Olea europaea*, L) are produced around the world, with the average consumption of this industrial product being 2.855.000 tonnes in 2022 according to the International Olive Oil Council (IOC). The countries with the highest production were Spain, Argentina, Morocco, Greece and Turkey, and the countries with the highest consumption were Egypt, Turkey, Algeria, the United States and Spain. This last country consumed 2.3% more than the previous year, reaching a total consumption of 185,000 tonnes.

There are different styles of table olive but, at industrial level, Spanish-style and Californian-style are the main table olive elaboration processes. The difference between them is that Californian-style—so-called black—table olives are submitted to alkaline and air treatment for bitterness and to oxidate the product. Table olives submitted to both processing styles are composed of monounsaturated fatty acids and other minor constituents, such as tocopherols and phenolic compounds [1]. In this sense, the black colour of Californian-style black olives induces the oxidation and polymerisation of phenols and, therefore, the phenol content of this type of olive is lower [2]. These olives are also subjected to thermal sterilisation treatment to ensure their microbiological stability between 121 and 126 °C [3].

Currently, Spanish-style table olives are stuffed with different herbs or spices that give them characteristic aromas, leading to good acceptance among consumers as they are more attractive for them. Furthermore, the main advantage of this method is that it is cheaper than using natural fillers. A wide variety of different types of stuffing, such as peppers, anchovies, garlic, almonds, etc., are normally used for green olives at the industrial level, and a very small amount of filler is added, entailing a reduction in production costs. In the table olive industry, it is very common to use hydrocolloids for stuffing to give the desired aroma and flavour. There is also the possibility of using fillings with natural food paste, but the disadvantage is that they are more expensive than hydrocolloid fillings [4]. However, Californian-style black olives with flavoured stuffing are not yet marketed, possibly due to the fact that the sterilization treatment significantly reduces the positive odour of the final product. Although this treatment affects the sensory characteristics of table olives, it must be applied in order to obtain a totally microbiologically stable product, since the final product has a neutral pH with low acidity and salinity.

Furthermore, the sterilisation treatment can lead to uncharacteristic aromas and flavours in table olives, such as cooking or burning effects [5]. Moreover, the high temperature of the sterilisation process may cause the loss of healthy compounds, such as phenols, due to the thermal degradation of the nutritional components. Other researchers have highlighted that sterilisation also affects antioxidant compounds and vitamins by causing their oxidative degradation [6].

According to Royal Degree 679/2016 [7] on the quality standard for table olives, olives can be classified according to their physical defects in different categories: extra, select and standard. For this classification, it is necessary to carry out a sensory analysis to determine whether the defects are due to strange smells, fermentation or lack of flavour. This must be carried out by well-trained and validated tasters.

The International Olive Council (IOC) recently published a recommendation related to sensory evaluation of table olives by panellists [8]. Sensory analysis is carried out by a tasting panel made up of experts; however, setting up a panel is usually costly and time-consuming and requires specialists who know how to evaluate the sensory qualities of food [4]. Alternatively, chromatographic analysis may be used to identify volatile organic compounds related to positive and negative aromas in olives, making it possible to characterize the product according to its commercial category [9,10].

There are electronic devices that can help the tasting panel evaluate olives based on their quality. This is the case for the E-nose electronic sensory device, which is used to discriminate between the aroma profiles of samples for different foods. Other authors have successfully used the E-nose to control the decrease in taste and quality for coffee beans [11], tomatoes [12] and even virgin olive oil [13]. In addition, the E-nose has been used to classify Spanish-style table olives into the extra, first and second categories [9] and even for the classification of sparkling wines after subjecting them to different thermal treatments and using different yeasts during the elaboration process [14]. This instrument is a low-cost method that allows rapid measurements to be obtained compared to the traditional methods, such as the chromatography instruments described above. E-noses could be used as a support for tasting panels.

Therefore, stuffed Californian-style black olives that provide a pleasant characteristic aroma would be interesting as an innovative product to be placed on the market. Thus, the aim of this work was to classify Californian-style black olives that underwent different sterilisation treatments in the extra, select and standard categories. The classification was based on a tasting panel, phenolic composition, an E-nose and volatile organic compounds. In addition, we intended to establish relationships between the aromatic and phenolic compounds obtained and the electronic device in order to develop predictions that would allow us to quantify certain parameters of the final product.

## 2. Materials and Methods

### 2.1. Reagents

For the table olive elaboration process, the following reagents were used: salt, sodium chloride, sodium hydroxide and ferrous gluconate (purchased from Sigma-Aldrich (St. Louis, MO, USA)); calcium chloride (Tetra Chemicals (Helsingborg, Sweden)); and acetic acid (Panreac (Applichem, Darmstadt, Germany)). Olives were stuffed with hydrocolloids and sodium alginate (Saporepuro, Torino, Italy), as well as guar gum (Saporepuro, Torino, Italy). The herbs and ingredients for the hydrocolloid preparation were purchased from a local store.

### 2.2. Raw Material

**Figure 1:**
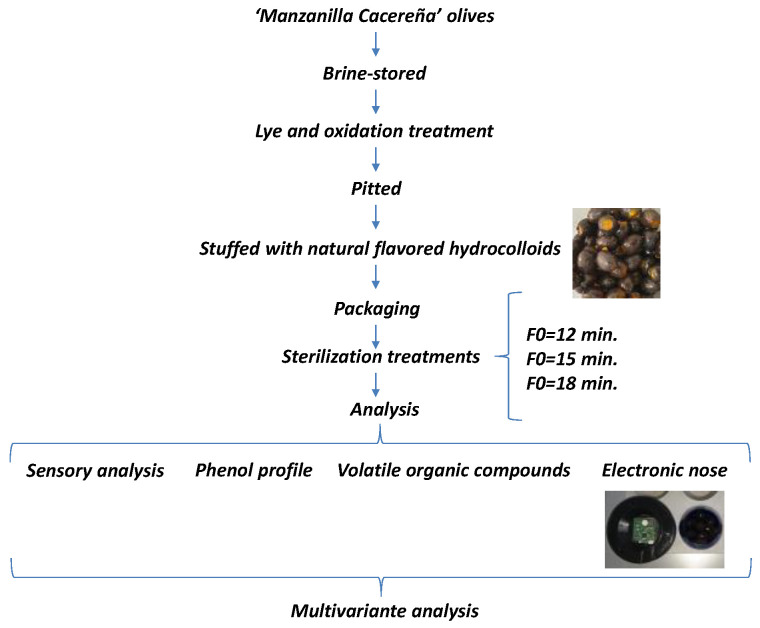
Diagram of the experimental design.

Table olives (*Olea europaea* L.) were harvested at a CICYTEX-collaborating farm in the north of Extremadura (southwest of Spain). The variety used was “Manzanilla Cacereña”, harvested at the green-yellow state of maturation during the olive-growing season in 2021/2022. Olives were stored in 10.000 kg capacity tanks in triplicate for 4 months with a private company. They were preserved in a solution of water with 1.5% acetic acid and 8.0% salt. Then, the Californian-style black olives were produced at the industrial level. A diagram of the experiment is shown in Figure 1.

### 2.3. Californian-Style Black Olive Production Process

The production process was carried out following the methodology proposed by Sánchez et al. [2]. After the conservation period, the olives were washed with water spray and immersed in water for 1 h. Subsequently, the liquid was removed and sodium hydroxide (3% p/v) was added to the olives. The fruits were also oxidated by applying artificial air. When the olives attained a black colour and were without bitterness, the pH inside the fruit was neutralized to 7 units after repeated washing and adding lactic acid. At this time, ferrous gluconate was added and olives were allowed to rest without the application of artificial air for 5 h. When the olives acquired a shiny black colour, they were ready to be stuffed with the corresponding aroma.

### 2.4. Hydrocolloid Preparation

After the Californian-style elaboration process, the olives were manually pitted and stuffed with hydrocolloids flavoured with natural spices: five cloves of garlic, one tablespoon of cumin, two tablespoons of sugar, one tomato, one chilli pepper, one red pepper, two tablespoons of sweet paprika, half a glass of vinegar, salt and one tablespoon of extra virgin olive oil. For the preparation, 20 g of salt was dissolved in a 1 litre container. Subsequently, the crushed and chopped garlic and the mixtures of aromatic spices were added. The tomato and pepper were crushed with a blender and added to the initial solution. Finally, the oil and vinegar were added. The final volume prepared was 1 L. This mixture was macerated for 24 h and refrigerated. The mixture was centrifuged at 21,036× *g* to obtain the flavoured liquid extract. This liquid was stored in a refrigerator at 4 °C before stuffing the olives.

A total of 200 mL of the flavoured liquid was mixed with sodium alginate and guar gum (2:1 ratio). A commercial mixture was used until the mix had a snail mucus texture. After that, the olives were manually filled with 10 mL of the mixture using a syringe. The stuffed olives were immersed in 4% calcium chloride solution for 24 h. The solution was removed and the olives were washed with water for 2 min. The brine solution was prepared by adding 2.0% sodium chloride, 0.5% calcium chloride and 0.015% ferrous gluconate. This solution was added to the stuffed olives in 150 g capacity cans. The assays were performed in quintuplicate.

### 2.5. Accumulated Lethality Values

Next, the cans were submitted to different sterilization processes and the accumulated lethality (F0) was calculated with the method proposed by Sánchez et al. [2]. This value corresponded to the time required to reduce the initial microbial load of the cans. The thermal treatments applied were 12, 15 and 18 min. Briefly, for each of the sterilization treatments, the cans were monitored with temperature sensors inserted into them. When the desired thermal treatment temperature and time were reached for each treatment, the F0 value was calculated. Subsequently, the sterilized cans with natural stuffed olives and brine solution were stored at room temperature and in the dark until the analysis was carried out. The assays were performed in quintuplicate.

### 2.6. Analysis

#### 2.6.1. Sensory Analysis

Californian-style black olives stuffed with flavour hydrocolloids and submitted to different thermal sterilization treatments were evaluated for their sensory qualities by a group of expert tasters who usually evaluate table olives in different research projects. The panellists were previously trained following the established recommendations by the IOC [8]. The tasting panel included eight panellists of both sexes aged between 30 and 60 years old. The sensory analysis was carried out in the CICYTEX research centre in a specific tasting room with sensory booths. To test whether the tasters were able to differentiate between the different categories of stuffed Californian-style black olives, a triangular test was carried out. This test consisted of comparing two samples from the same category and one from a different category to see if there were sensory differences between them. The tasters had at their disposal apple pieces and mineral water to rinse their taste buds between samples.

The sensory evaluation was carried out in a tasting room, and three olives for each sample were placed into standard tasting glasses with 10 mL of the brine solution. The standard glasses were covered with a watch glass and placed on a heating block at 25 ± 2 °C [8]. Descriptive profiles were assessed using a test sheet where each attribute was scored. Olfactory aspects were also assessed in this study. Samples were sensorially classified into different categories according to the defect predominantly perceived (DPP) by the tasting panel: extra (DPP ≤ 3), select (3 < DPP ≤ 4.5) and standard (4.5 < DPP ≤ 7.0). For the purposes of this experiment, fruity odour was classed as a positive attribute and burn defects were classed as a negative attribute to evaluate the aroma intensity of the olives. Tasters had to fill a structured 10 cm scale to quantify the odour. Data from the sensory analysis were expressed as mean values and they were considered reliable when the coefficient of variation (CV) was less than 20% [8]. The samples were evaluated over a week with two sessions per day.

#### 2.6.2. Phenol Profile Analysis

The phenol profile analysis was undertaken following the methodology proposed by Cabrera-Bañegil et al. [15]. Two g of the crushed and homogenised samples was used. This was extracted with 10 mL of methanol containing 2 mM NaF. Samples were placed in an ultrasonic bath for 30 min (P-selecta ultrasonic bath, mod 516, Barcelona, Spain). Everything obtained was centrifuged for 10 min at 1677× *g* at 4 °C (Thermo Scientific Sorvall Legend XT/XF centrifuge with a F13-14x50c carbon fiber rotor, Thermo Fischer Scientific, Waltham, MA, USA). Finally, the extracted supernatant was passed through a 0.22 µm nylon syringe filter (FILTER-LAB, Barcelona, Spain) and HPLC was performed.

#### 2.6.3. Volatile Organic Compounds

The volatile organic compounds in the Californian-style black olives were analysed by injecting the samples previously absorbed with a polydimethylsiloxane/divinylbenzene (PDMS/DVB) Stable-Flex fiber (65 µm, Supelco, St. Louis, MO, USA) in a Bruker Scion 456-GC triple quadrupole gas chromatograph with a DB WAXETR capillary column [9]. A total of 2 g of the crushed olives from each of the treatments was weighed in a 15 mL glass vial with 7.0 mL of 30% (*w*/*v*) NaCl. After that, the absorbed compounds were desorbed into the equipment at 250 °C for 15 min. The compounds were identified from matching mass spectra with the NIST 2.0 MS standard library.

#### 2.6.4. Electronic Device

A group of researchers from the University of Industrial Engineering of Extremadura designed the handmade E-nose used. This device contained four gas sensors, the signal processor, the controller of the system and the communication module. For each sample, an odour pattern was generated by the E-nose. More information about this device can be found in a research paper published by Arroyo et al. [16].

The measurements of the table olives were carried out according to the recommendations for sensory analysis from the International Olive Council [8]. Table olives were introduced into standard glasses with three olives in each, and the glasses were placed over the heating plate at 25 °C. A watch glass was placed on top of each glass. The E-nose was placed in the sample beaker for 60 s and the signals were recorded. Next, for desorption, the E-nose was placed in the glass without a sample. For each sample, 11 measurements were performed. All data obtained from the measurements with the E-nose were sent to a smartphone device via Bluetooth.

### 2.7. Multivariate Analysis

Radial diagram can be used to show the signals emitted by E-nose sensors. To obtain this diagram, the E-nose data were normalised using the formula: (X_i_ − X_Min_)/(X_Max_ − X_Min_), where X_i_ is the experimental measurement for sample i; X_Min_ is the lowest experimental data series value; and X_Max_ is the highest experimental data series value.

After that, the data provided by the E-nose were also analysed with multivariate algorithms using principal component analysis (PCA), partial least square discrimination analysis (PLS-DA) and partial least squares (PLS). PCA was carried out to observe the differences between table olive categories [17]. The PLS-DA was performed to evaluate the ability to discriminate between samples according to the thermal treatment applied [18]. PLS was performed to quantify the defective aromas perceived by the panellists and the total phenol profile obtained using the E-nose. Data analysis was carried out in MATLAB version R2016b (MathWorks Inc., Natick, MA, USA) and PLS Toolbox version 8.2.1 (Eigenvector Research Inc., Wenatchee, WA, USA).

### 2.8. Statistical Analysis

A statistical analysis was undertaken using one-way ANOVA and Tukey’s test to determine significant differences between different treatments (*p* < 0.05). The statistic program used was SPSS 18.0 (SPSS Inc., Chicago, IL, USA). The same statistical analysis was undertaken for the triangular test. The results are presented as mean values and standard deviations.

## 3. Results and Discussion

### 3.1. Sensory Odour Characteristics of Californian-Style Black Olives Stuffed with Hydrocolloids

To evaluate the sensory differences between the different categories of Californian-style black olives stuffed with natural aromas, a triangular test was performed (Table 1). Other researchers have used this test for olives that underwent different calcium treatments to determine whether the tasters were able to discriminate between them [19]. According to the data obtained, the different samples could be clearly distinguished in the different tests. It should be noted that the extra category differed from the other two since the rate of success was 100%. However, the rates of success for the standard and select categories evaluated by tasters were 85% and 89%, respectively. Thus, practically all the samples were identified by the tasters according to their characteristic odours.

For this reason, a descriptive sensory evaluation of the positive attributes and the burning effect of the olives studied was carried out (Table 2). Californian-style black olives stuffed with natural hydrocolloids were classified into the extra, select and standard categories according to their sensory attributes. The extra category had the strongest positive aroma and the weakest burn aroma. It should be noted that the natural aromas added to the olives were clearly detected by the tasters despite the thermal sterilization treatments. This is a good result that could help producers implement healthy fillings in this type of olive. Despite these flavoured fillings, the tasters detected burn defects. This characteristic smell is related to the cooking effect resulting from sterilisation. The standard category had the strongest burn aroma, while the select category fell between the two categories above. In this sense, in agreement with [6], it was observed that uncontrolled sterilisation periods may be responsible for aroma defects. Again, the sterilisation treatment also caused the appearance of certain defects in the olives. The more intense the sterilization was, the greater the number of burn defects detected. In this case, the flavoured natural hydrocolloid aroma could have contributed to the fact that the burnt defects were not so intense in the various samples. We also classified the table olives according to the average intensity of the DPP using an electronic tongue [20].

### 3.2. Phenol Profiles of Californian-Style Black Olives Stuffed with Hydrocolloids

The phenol profiles of the Californian-style black olives stuffed with hydrocolloids were quantified (Table 3). As can be seen, a few varieties of phenolic compounds were identified in this type of olive. Californian-style black and green olives are two of the styles with the lowest phenol profiles compared to natural and even Spanish-style olives [21]. The main phenols in our study, independently of the category, were hydroxytyrosol, followed by tyrosol and oleuropein. Other phenols present in the olives were procyanidin B1, *p*-coumaric and verbascoside. The minor ones were vanillic acid, (-)-epicatechin and luteolin-7-*O*-glucoside. We have to highlight that the minor compounds were not detected in the lower-quality table olive categories. This was the case for the select and standard categories. Thus, the amount of phenols decreased when the quality of the table olives was lower, as can be seen from the total phenols, which is the sum of all phenols quantified in each category. The decrease in phenols in the lowest category could have been due to the fact that the standard olives, which presented the strongest burn defects, were submitted to a more aggressive thermal sterilization treatment. Phenols are thermolabile, so the high intensity of heat during the sterilization period could have triggered the degradation of these compounds. If we compare the content of phenols with results from other studies carried out by other researchers, it can be seen that the content in this study was slightly higher [22]. This could have been due to the fact that the flavoured hydrocolloid fillings increased the phenol content of the samples compared to other Californian olives.

**Figure 2:**
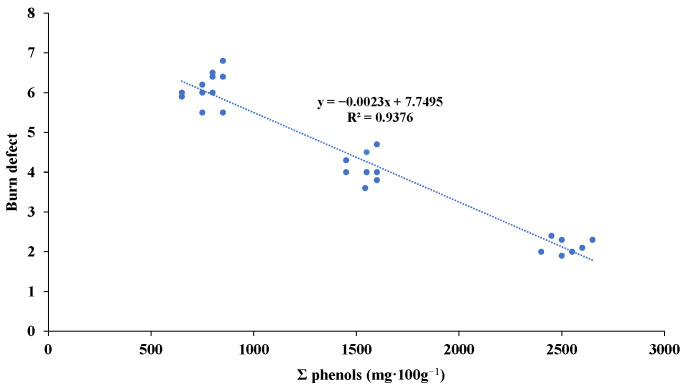
Linear regression for burn defects and Σ phenols in Californian-style black olive stuffed with hydrocolloids.

A correlation was established between the total phenolic compound content and the burn defects evaluated sensorially. As can be seen in Figure 2, a negative correlation was established, since the amount of phenols decreased when the burn defect was strong. High phenolic compound content was observed when the burn defect was very weak. This relationship helped us to verify that the intense thermal treatments applied to Californian-style black olives caused strong burn defects that significantly affected the contents of certain compounds with health benefits.

### 3.3. Discrimination of Olive Aroma Categories Using an Electronic Device

**Figure 3:**
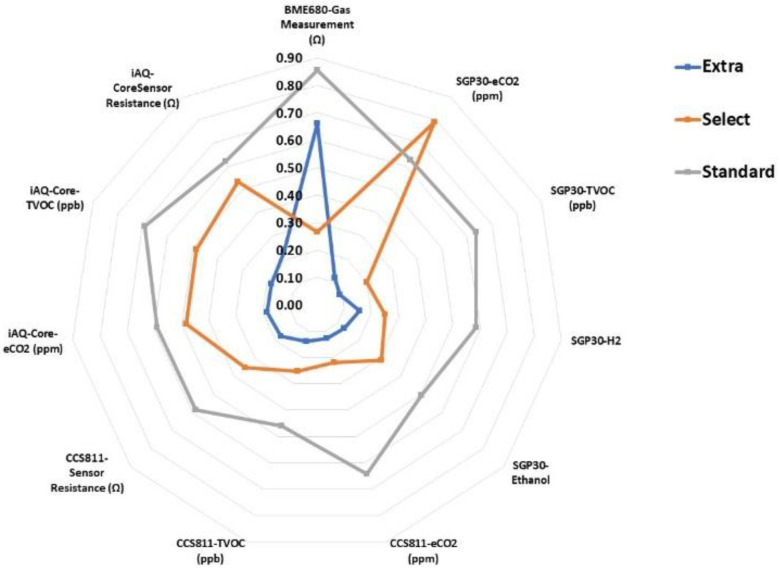
Radial plot of the sensor´s responses to Californian-style black olives stuffed with hydrocolloids. BME680: gas measurement (Ω); SGP30: eCO_2_ (ppm), organic volatile compounds (ppb), H_2_ and ethanol; CCS811: eCO_2_ (ppm), organic volatile compounds (ppb) and sensor resistance (Ω); iAQ-Core: eCO_2_ (ppm), organic volatile compounds (ppb) and sensor resistance (Ω).

The radial plot of the sensor’s responses to the aromas of Californian-style black olives with natural flavoured hydrocolloids is shown in Figure 3. As can be seen, each table olive category had a particular fingerprint that corresponded to each of the responses of the sensor to the aromas of the samples. In the standard samples, most of the sensor responses were stronger than the others because the values were near to 1, while for the extra category, the sensor responded with less intensity to the aromas of the samples, except for the BME680 gas measurement. Thus, we can deduce that the profiles of the aromas of each sample after analysis with the E-nose were completely different and could be distinguished by this electronic device.

**Figure 4:**
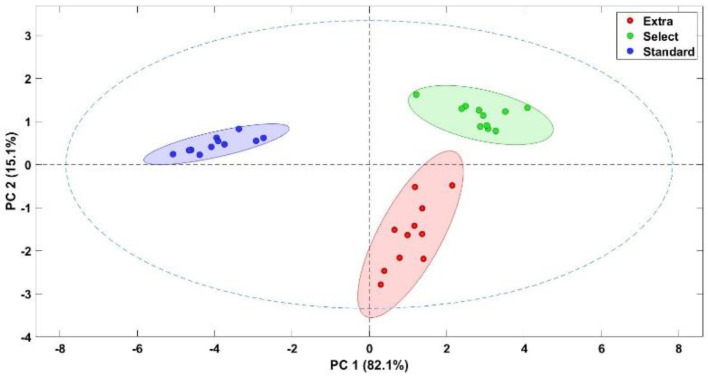
Score plot for the PCA analysis of Californian-style black olives stuffed with hydrocolloids.

Then, the 11 signals recorded by the sensors of the E-nose were reduced to two different components (Figure 4). The principal component analysis showed that the responses of the E-nose to the different samples from each category could be distinguished from each other. PC1 explained 82.1% of the variance while PC2 explained 15.1%. Thus, the odour profiles of the samples detected by the electronic device were enough to discriminate between samples of different categories. Data from the extra category occupy the right-hand side of the plot and correspond to positive PC1 scores, while the select and standard categories showed positive PC2 scores and are in the upper two quadrants of the figure. Thus, it can be seen that the different samples from the different categories could be distinguished.

Therefore, when good discrimination was observed in the previous PCA, another classification model, PLS-DA, was used to establish a predictive classification to determine the different classification categories for the table olives studied. Table 4 shows the results of the confusion matrix, which was able to correctly classify almost all the samples. Only 9.0% of the samples in the select category were classified in a different category (extra). This may have been due to the standard deviation itself during the measurement with the electronic equipment. However, the classification model was satisfactory since it was able to predict the category of Californian-style black olives with 90.9% accuracy.

Researchers [2,22] have classified Californian-style black olives subjected to five different thermal sterilisation treatments based on the aromatic profiles of the samples. These researchers reported a strong burn odour from the samples subjected to more aggressive thermal sterilisation treatments. In our case, such severe burn smells were not detected because, in our study, the olives were flavoured with a natural hydrocolloid filler that was able to mask the intense burn smell from the most intense thermal sterilisation treatments.

### 3.4. Aromas of Californian-Style Black Olives Stuffed with Hydrocolloids

**Figure 5:**
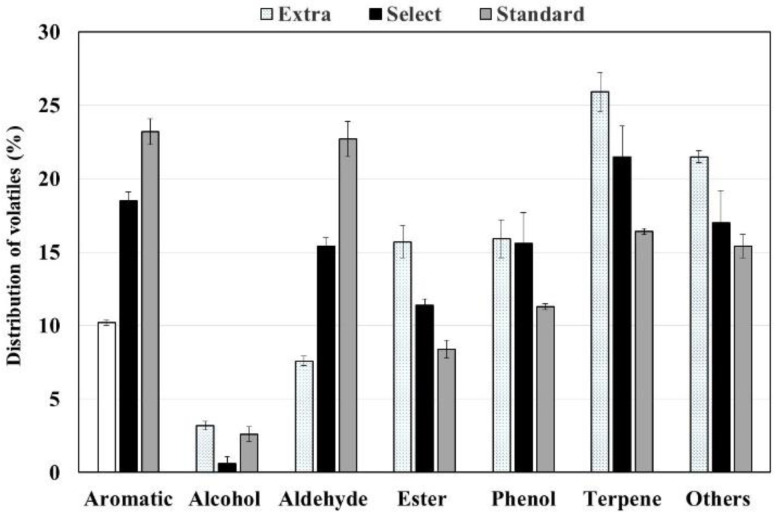
Distribution (%) of chemical families of VOCs in Californian-style black olives stuffed with hydrocolloids.

The Californian-style black olives stuffed with hydrocolloids presented a characteristic aroma for which seven different chemical families of volatile organic compounds (VOCs) were identified (Figure 5). Significant differences were observed in each of the families identified in relation to the qualities of the olives. Terpenes stood out as the most abundant family in extra quality olives, accounting for 25.9% of the total volatiles. This proportion of VOCs from the terpene family decreased to 21.5% for the select quality olives and showed a still lower proportion in standard quality olives, reaching only 16.4%.

Similarly, large aromatic differences were observed for the families of esters, phenols and the other VOCs in olives depending on their quality. In these cases, the most abundant volatiles were observed in the highest quality olives. Their proportions decreased in the select quality olives and even more so in the standard quality ones. Aromatic compounds and aldehydes were also abundant, especially in standard quality olives (23.2% and 22.7% of total VOCs, respectively). In these two families, the differences between the samples of different qualities appeared in the opposite way to those described above since they were more abundant in lower quality olives, where they exceeded 20%, and decreased to 10% or less in olives of higher quality. Finally, the family of alcoholic compounds had the lowest presence in all the samples and was found in very low proportions, especially in select quality olives (0.6%).

Different researchers have studied VOCs in olives. Martín-Tornero et al. [22] subjected olives to thermal treatments and found that aldehydes and other groups of VOCs increased when the thermal treatment was enhanced. The group of alcohols was underrepresented in these olives. On the other hand, the contents of esters and phenols decreased when the sterilisation treatment was intensified. Previous studies have also associated the quality of olives with the presence of terpenes. Perestrelo et al. [23] found that the volatile profile of olive oil was slightly influenced by a maceration process at room temperature (20 ± 2 °C) for 15 days. The predominant differences were observed for the group of terpenoids, since some were only identified in the flavoured olive oils, while others showed increases with the maceration process. Furthermore, Montero-Fernández et al. [24] studied the masking of sensory defects with carao (*Cassia grandis*) in flavoured stuffed olives. Carao is a Native American plant rich in iron. The incorporation of carao in the processing of Californian-style black olives added a pleasant, sweet odour but also introduced negative sensory characteristics. such as cheese, fermented and metallic flavours/aromas. However, these aromas caused by *C. grandis* could be neutralized by filling the olives with mojo picón flavoured hydrocolloids, thus enhancing the commercial grade of the table olives. Mojo picón is a commercial flavouring. The addition of fresh carao to the olives resulted in higher levels of hydrocarbons and oxygenated compounds. However, carboxylic acids and alcohols decreased. When mojo picón was used to cover up the smell of carao at a 2.0% concentration, it was observed that terpenoids greatly increased, while hydrocarbons, oxygenated compounds and alcohols decreased. Conversely, when these researchers added mojo picón at a 4.0% concentration, acid derivatives were found in low amounts and carboxylic acids increased but terpenoids decreased [24].

The main VOCs found in each of the olives are described below according to the different categories of quality (Table 5). In general, the samples had varied and complex volatile chemical compositions with a wide variety of odours. The most abundant compound in the extra quality olive samples was creosol (19.2%), which presents a spicy, clove and vanilla aroma, as well as a medicinal phenolic odour [25,26]. Further, the diallyl disulphide (12.1%) compound, characterized by a strong garlic aroma, was identified in the highest quality samples [27]. In addition, gamma-terpinene (11.6%), an important component that provides a refreshingly tropical herbaceous-citrus smell, was detected in the extra quality samples [28]. Furthermore, *p*-cymene (10.3%), which has a mild and pleasant smell of wood and citrus [29], and beta-pinene (9.4%), a compound with a woody, dry and resinous green pine aroma, were identified [30]. The olive samples for the highest-quality commercial category also contained cyclohexanecarboxylic acid; ethyl ester (6.6%), which is perceived as a fruity and cheesy wine smell [31]; and, in very similar proportions, copaene (6.5%), which has an earthy touch similar to boiled potato [32]. Styrene (5.3%) was also identified, presenting a sweet, balsamic and floral odour with a plastic touch [33], together with *p*-xylene (5.1%), which presents a sweet, aromatic odour [34], and 3-hexen-1-ol, acetate (3.9%), which has a fresh, sweet, green apple or herbaceous odour [35]. Although at a very low concentration, the compound octan-1-ol (1.7%) was specific to this sample from the extra quality olives and stood out for its fresh and citric aroma [36].

Regarding the aromas identified in the select quality olives, creosol again stood out (18.4%) with a spicy aroma of cloves and hints of vanilla [25]. It was followed by the compound benzoaldehyde (12.8%), which has a strong, sharp, sweet and bitter almond aroma [37], and 4-ethenylpyridine (6.7%), which is noted in the literature for its pungent and unpleasant odour [22]. Finally, diallyl disulphide was also identified at a proportion of 6.5%, which is notable for its strong garlic odour [38]. The rest of the compounds were held in common between the extra and standard quality samples, although the concentrations were different and generally intermediate, as indicated in Table 2.

The most characteristic aromatic compound in the standard quality olive samples was benzaldehyde (21.0%), which has a strong, sharp and sweet odour with hints of bitter almond. 4-Ethenylpyridine was also quite common in these samples (13.3%), with a pungent and unpleasant odour [22]. 3-Methyl-pyridine (6.1%) contributed an aroma of earth and hazelnut, sweet and not unpleasant [39]. Beta-farnesene (5.8%) has a woody, citrusy and herbal aroma and is sweet to the nose [40]. Finally, 2,4-dimethylhexane (4.8%) was also present, although in small proportions, and it has an unpleasant odour [22].

Finally, it should be noted that some compounds were directly related to the higher quality olives, increasing their concentrations progressively in the select and extra quality olives. Among these compounds were creosol, diallyl disulphide, gamma-terpinene, *p*-cymene and beta-pinene, results that agree with those obtained by Sánchez et al. [9] in their study on table olives, these being the main volatile components in samples without defects. In contrast, compounds such as benzaldehyde, 4-ethenylpyridine, 3-methyl-pyridine and beta-farnesene were more abundant the lower quality samples. It should be noted that octan-1-ol only appeared in the extra quality samples, although in a very low concentration, while 3-methylpyridine was not detected in samples of the highest commercial quality.

4-Ethenylpyridine, benzaldehyde and 2,4-dimethylhexane had great influences after the application of heat treatment, increasing in content. These volatile compounds have unpleasant odour descriptors [22]. In addition, the compounds responsible for other fruit aromas, such as ethyl ester cyclohexanecarboxylic acid and creosol, decreased in content. These results agree with the variations in the VOCs observed in the samples of different qualities, since higher proportions of these unpleasant compounds were found in the samples of lower quality. Similarly, Sánchez et al. [41], when comparing defects in healthy and defective green olives from Zapateria, found similar VOCs to those identified in this study. The aromas of these compounds were associated with fresh fruit, citrus, herbs and green onions. These results agree with those observed here, since all of them appeared in higher proportions in the higher quality samples.

López-López et al. [42] investigated the impacts of various stages of ripe black olive processing on the VOCs of two olive cultivars. They found that the preservation step enhanced the volatile profiles of the olives, primarily with regard to ethyl acetate, methyl acetate and ethanol. The darkening step resulted in the total or partial elimination of 55.0–65.0% of the volatiles identified before this step. About 70.0% of the VOCs in the olives were compounds related to the sterilisation treatment. The dominant volatiles were benzaldehyde, dimethyl sulphide and ethyl acetate. Benzaldehyde appeared in a higher proportion in lower quality olives but dimethyl sulphide and ethyl acetate were not present in any of them. Similarly, Sabatini et al. [43] obtained the characteristic volatile organic compounds of table olives (*Olea Europaea* L.) using gas chromatography.

### 3.5. Burn Defect and Phenol Profile Quantification Using an Electronic Device

**Figure 6:**
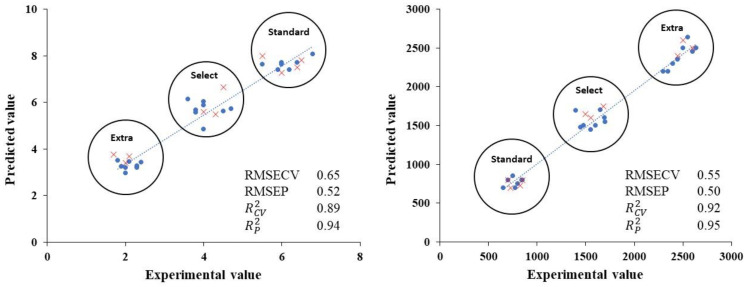
Experimental values compared to PLS cross-validation predictions (●) and validation set predictions (x) for burn defects in aromas perceived by panellists (**left**) and total phenol profiles (**right**).

A partial least squares (PLS) model was established to quantify the burn defects in the odours perceived by the panellists and the total phenol profiles (Σ phenols) for the samples from the different table olive categories (Figure 6). The results showed good relationships between both variables studied and the electronic device. It was found that the different thermal treatments applied to the samples were related to the aromatic profiles and, indirectly, to the representative phenolic profiles of each of the categories studied. These results were reflected in the parameters in the models established. Thus, the $R_{CV}^{2}$ values for the burn defects and total phenol profiles for the models established were 0.89 and 0.92, respectively. The results for the RMSECV values were 0.65 for burn defects and 0.55 for total phenols. The model was also validated with external samples, obtaining $R_{P}^{2}$ values of 0.94 for burn defects and 0.95 for total phenols. The RMSEP values were less than 0.52 in both cases.

The characteristic aromas of these olives could be predicted with the electronic device in a fast and efficient way. This model also made it possible to satisfactorily predict the total phenol profiles of the samples studied. This would be interesting for the prediction of the content of substances with health properties. In fact, higher phenol content was observed in the extra category and, therefore, these olives would be healthier. In the scientific literature, relationships between electronic devices and aromatic parameters of samples have been established. Sánchez et al. [2] predicted the strength of the odour defects in Californian-style black olives with no flavoured filler added. Another study [22] established an indirect prediction model for an electronic device and the quantity of acrylamide, although this toxic substance does not smell. However, other researchers have used this method for the classification of roasted coffee samples into different categories depending on their quality with an E-nose [44].

## 4. Conclusions

The sterilisation treatments induced burn defects in Californian-style black olives despite the fact that the olives were stuffed with natural fillings. Furthermore, the thermal sterilisation treatments decreased the amount of phenols in these olives. This made it possible to classify the products according to the different commercial categories established in the IOC regulations. Moreover, the electronic equipment developed made it possible to classify the olives according to their sensory quality. This was because the characteristic volatile organic compounds in the samples varied between categories. The volatiles present in the highest quality samples (extra) were creosol, diallyl disulphide, gamma-terpinene and *p*-cymene, while in the worst quality samples (standard), benzaldehyde, 4-ethenylpyridine, 3-methyl-pyridine and 2-4-dimethylhexane were identified. Finally, it was possible to quantify the burn defects and the total phenol content in the samples studied with the E-nose. This result is interesting since measurement with the electronic device is fast and does not require a high degree of specialization and the results are very reliable and safe.

## Figures and Tables

**Table 1:** Results of the triangular sensory analysis test for Californian-style black olives stuffed with hydrocolloids.

	Extra	Select	Standard	
**Extra**	---	*p* < 0.05 *n* = 5 100%	*p* < 0.05 *n* = 5 100%	**Extra**
**Select**	*p* < 0.05 *n* = 5 100%	---	*p* > 0.05 *n* = 5 85%	**Select**
**Standard**	*p* < 0.05 *n* = 5 100%	*p* > 0.05 *n* = 5 89%	---	**Standard**

*n*: Number of samples analysed.

**Table 2:** Sensory aroma attributes of Californian-style black olives stuffed with hydrocolloids.

Variety	DPP Classification	Positive Aroma	Burn Defect
“Manzanilla Sevillana”	Extra	4.3 ± 0.5 a	2.1 ± 0.2 c
	Select	3.2 ± 0.5 b	4.1 ± 0.3 b
	Standard	1.9 ± 0.3 c	6.1 ± 0.4 a

Results are expressed as means ± SD of three sample replicates. Different lowercase letters mean significant differences between the different commercial categories (one-way ANOVA followed by Tukey’s test, *p* < 0.05)

**Table 3:** Chemical composition of Californian-style black olives stuffed with natural fillings.

Phenolic Profile (mg·100 g^−1^)	Extra	Select	Standard
Hydroxytyrosol	1594.5 ± 76.7 ^b,C^	1104.1 ± 17.4 ^b,B^	551.8 ± 26.2 ^b,A^
Tyrosol	405.7 ± 7.1 ^c,C^	235.9 ± 19.3 ^c,B^	127.0 ± 6.3 ^c,A^
Procyanidin B1	53.8 ± 3.9 ^e,C^	33.0 ± 6.6 ^e,B^	15.5 ± 1.0 ^e,A^
Vanillic acid	8.8 ± 0.6 ^h^	n.d.	n.d.
(-)-Epicatechin	5.5 ± 0.1 ^i^	n.d.	n.d.
Oleuropein	311.3 ± 12.0 ^d,C^	185.9 ± 8.9 ^d,B^	72.3 ± 1.5 ^d,A^
Luteolin-7-*O*-glucoside	7.9 ± 0.7 ^h,B^	4.9 ± 0.3 ^h,A^	n.d.
Apigenin-7-*O*	10.1 ± 0.6 ^g,B^	7.4 ± 0.2 ^g,A^	n.d.
Verbascoside	11.5 ± 1.2 ^g,B^	8.9 ± 0.4 ^g,A^	n.d.
*p*-Coumaric acid	28.1 ± 1.6 ^f,B^	19.7 ± 0.8 ^f,A^	n.d.
Σ phenols	2437.4 ± 15.7 ^a,C^	1599.9 ± 6.7 ^a,B^	766.6 ± 30.4 ^a,A^

n.d.: Not detected. Different lowercase letters indicate statistically significant differences between treatments (one-way ANOVA followed by Tukey’s test, *p* < 0.05). Different capital letters mean significant differences between the different commercial categories for each phenol identified (one-way ANOVA followed by Tukey’s test, *p* < 0.05).

**Table 4:** Confusion matrix obtained through PLS-DA for Californian-style black olives stuffed with hydrocolloids.

Predicted Class			
Real Class	Extra	Select	Standard
Extra	33.3	9.0	0
Select	0	24.3	0
Standard	0	0	33.3

Values are expressed as percentages of the samples studied.

**Table 5:** Relative contents of volatile compounds (mean % (*n* = 3)) obtained from Californian-style black olives stuffed with hydrocolloids submitted to different sterilisation treatments.

	RT	DPP Classification		
	(min)	Extra	Select	Standard
2,4-Dimethylhexane	6.7	0.5 ± 0.0	2.7 ± 0.5	4.8 ± 0.1
3-Methylpyridine	10.0	n.d.	2.6 ± 0.6	6.1 ± 0.2
*p*-Xylene	10.2	5.1 ± 0.1	3.7 ± 2.4	1.7 ± 0.2
Styrene	11.3	5.3 ± 0.3	4.1 ± 0.4	3.3 ± 0.3
Benzaldehyde	15.3	2.5 ± 0.4	12.8 ± 2.0	21.0 ± 2.5
Beta-pinene	15.5	9.4 ± 0.5	7.2 ± 1.5	5.0 ± 0.8
4-Ethenylpyridine	15.6	1.6 ± 0.1	6.7 ± 1.2	13.3 ± 0.4
3-Hexen-1-ol, acetate	17.5	3.9 ± 0.4	2.5 ± 0.2	1.8 ± 0.4
Acetic acid, hexyl ester	17.9	2.2 ± 0.2	1.9 ± 0.2	1.3 ± 0.2
*p*-Cymene	18.1	10.3 ± 0.6	7.9 ± 0.3	6.1 ± 0.1
Gamma-terpinene	19.9	11.6 ± 0.4	10.3 ± 0.2	7.1 ± 0.2
Diallyl disulphide	21.1	12.1 ± 0.3	6.5 ± 0.4	2.5 ± 0.4
Octan-1-ol	21.2	1.7 ± 0.5	n.d.	n.d.
Beta-farnesene	23.1	1.6 ± 0.1	2.6 ± 0.1	5.8 ± 0.1
Cyclohexanecarboxylic acid, ethyl ester	24.1	6.6 ± 1.2	4.8 ± 1.3	4.1 ± 1.2
Creosol	27.2	19.2 ± 0.8	18.4 ± 2.3	12.5 ± 1.1
Copaene	35.3	6.5 ± 1.1	5.3 ± 0.4	3.7 ± 1.1

RT, retention time; n.d., not detected.

## Data Availability

The authors confirm that the data supporting the findings of this study are available within the article and the raw data that support the findings are available from the corresponding author upon reasonable request.
